# Supplementary material for: Content comparison of unmet needs self-report measures for lymphoma cancer survivors: A systematic review
Source: PLoS One. 2023 Dec 15;18(12):e0290729. doi: 10.1371/journal.pone.0290729 (PMC10723710; doi:10.1371/journal.pone.0290729)
Supplement: S1 Table — (DOCX) [file pone.0290729.s001.docx]

S1 Table. Excluded instruments

| Exclusion reason | Instrument name |
| --- | --- |
| Wrong population (n = 2) | - Support Persons Unmet Needs Survey (SPUNS) - Childhood Cancer Survivor Study (CCSS) |
| Wrong outcome/concept (n = 7) | - Memorial Symptom Assessment Scale Short Form (MSAS-SF) - Posttraumatic Growth Inventory (PTGI) - Posttraumatic Stress Disorder Checklist - Civilian Version (PCL-C) - Charlson Comorbidity Index (CCI) - Profile of Mood States (POMS) - The Princess Margaret Hospital Satisfaction with Doctor Questionnaire (PMH-PSQ-MD) - Body Uneasiness Test (BUT) |
| Too specific (n = 9) | - Functional Assessment of Chronic Illness Therapy – Fatigue (FACIT-F) - Functional Assessment of Cancer Therapy – Bone Marrow Transplant (FACT-BMT) - Functional Assessment of Cancer Therapy - Anaemia (FACT-An) - European Organisation for Research and Treatment of Cancer Quality of Life Questionnaire – Information Module (EORTC QLQ - INFO25) - Hospital Anxiety and Depression Survey (HADS) - Depression, Anxiety and Stress Scale (DASS-21) - Medical Outcomes Study Social Support Survey (MOS-SSS) - Hamilton Anxiety Rating Scale (HAM-A) - Appraisal and Life Threat and Treatment Intensity Questionnaire (ALTTIQ) |
| Not validated (n = 1) | - Houts et al Service Need Items (not validated) |
