# Supplementary material for: Content comparison of unmet needs self-report measures for lymphoma cancer survivors: A systematic review
Source: PLoS One. 2023 Dec 15;18(12):e0290729. doi: 10.1371/journal.pone.0290729 (PMC10723710; doi:10.1371/journal.pone.0290729)
Supplement: S1 Appendix — (DOCX) [file pone.0290729.s003.docx]

Appendix A. The reliability and validity of unmet needs and quality of life instruments used with adult lymphoma survivors

| **Unmet Needs Measure** | **Reliability** | **Validity** |
| --- | --- | --- |
| CaSUN [1] | Internal consistency: Cronbach’s alpha value = 0.96 for the multidimensional total scale and ranged between 0.78 and 0.93 for subscales.  Reproducibility: Time interval = approximately three weeks. Mean test-retest reliability (Kappa coefficient) score = 0.13. | Content validity: Based on previous qualitative research and literature review. Quantitative survey methods.  Construct validity: Exploratory factor analysis, five factors. |
| SCNS-SF34 [2] | Internal consistency: Cronbach’s alpha values ranged from 0.86 to 0.96 for subscales. | Content validity: Derived from the original SCNS-LF59, the origin of the construct is not clearly described. Quantitative survey methods.  Construct validity: Confirmatory factor analysis, five factors.  Convergent validity: correlation with three other measures assessing psychosocial well-being, Distress Thermometer, Hospital Anxiety and Depression Scale and EORTC QLQ-C30 (𝑟 = 0.48-0.56). |
| SUNS [3-5] | Internal consistency: All Cronbach’s alpha values were greater than 0.9 for subscales.  Reproducibility: Time interval = around 28 days. High item test-retest reliability was stated without data shown [4]. Weighted Kappa coefficients between item responses from Time 1 and Time 2 ranged from 0.25 to 0.76 (mean = 0.58; SD = 0.09) [5].  Cross-cultural reliability: face and content validity for Canadian and Australian haematological cancer patients [4]. | Content validity: Literature review, cancer survivor input, professional input, and a pilot test with feedback.  Construct validity: Exploratory factor analysis, five factors.  Convergent validity: correlation with all three subscales of the Depression Anxiety and Stress Scale (range 0.44 – 0.73). |
| SF-SUNS [6, 7] | Internal consistency: All Cronbach’s alpha values were greater than 0.85 for subscales [6]. Cronbach’s alpha values ranged from 0.65 to 0.94 for subscales [7].  Reproducibility: Time interval = 5 days. Test-retest reliability (intraclass correlation with 95% confidence interval) scores = 0.45 – 0.74 [7]. | Content validity: Derived from the SUNS.  Construct validity: Exploratory factor analysis, five factors. Intraclass correlations with the original domains of the SUNS were greater than 0.9, closely reflecting the original structure [6]. |

| **Quality of Life Measure** | **Reliability** | **Validity** |
| --- | --- | --- |
| EQ-5D-5L [8] |  | Content validity: Derived from the EQ-5D-3L. Focus groups were used to investigate the face and content validity of the new version. |
| EORTC QLQ-C30 [9, 10] | Internal consistency: Cronbach’s alpha values were 0.54-0.86 for pre-treatment and 0.52-0.89 for post-treatment.  Reproducibility: Test-retest reliability (Pearson’s correlation coefficient) scores = 0.82-0.91 [10]. | Content validity: Derived from the original EORTC QLQ-C36. Quantitative survey methods. |
| FACT-G [11] | Internal consistency: Cronbach’s alpha values ranged from 0.65-0.89 for subscales.  Reproducibility: Time interval = 3 to 7 days. Test-retest reliability (intraclass correlation) scores = 0.82 – 0.92. | Content validity: interviews with cancer patients and oncology professionals for item generation, item review/reduction and pilot test/evaluation.  Convergent correlations: correlations performed with multiple tools, highest with the Functional Living Index-Cancer (𝑟 = 0.79) and lowest with the Marlowe-Crowne Social Desirability Scale (𝑟 = 0.22). |
| FACT-Lym [12] | Internal consistency: Cronbach’s alpha values were 0.70-0.95 at all time points.  Reproducibility: Time interval = three-time points (baseline, 3-7 days, and 8-12 weeks). Test-retest reliability (intraclass correlation) scores = 0.61-0.87. | Content validity: item generation using semi-structured interviews with cancer patients and oncology professionals, item review/reduction and pilot test/evaluation.  Convergent validity: correlation between the FACT-Lym and other FACT scales with the Profile of Mood States total score (range = -0.42 to 0.68), SF-36 mental (𝑟 = 0.48, 𝑃 < 0.001) and physical (𝑟 = 0.62, 𝑃 < 0.001). |
| IOC [13] | Internal consistency: Cronbach’s alpha values were 0.67-0.89 for subscales. | Content validity: qualitative methods, nurse and social worker input, content analysis, pilot test. |
| IOCv2 [14, 15] | Internal consistency: Cronbach’s alpha values were 0.76-0.89 for breast cancer participants and 0.59-0.91 for non-Hodgkin lymphoma participants [15]. | Content validity: derived from the original IOC.  Construct validity: De novo exploratory factor analysis.  Concurrent validity: lack of correlation between the IOCv2 positive scale and the FACT-G, FACT-Lym and SF-36, showing the IOCv2 content is distinct from these scales. |
| QoL-CS [16] | Internal consistency: Cronbach’s alpha values were 0.71-0.89 for subscales.  Reproducibility: time interval = approximately two weeks. Overall test-retest reliability was 0.89. | Content validity: in-depth interviews, quality of life researcher and nurse input.  Construct validity: Exploratory factor analysis.  Concurrent validity: the overall correlation with the FACT-G was 0.78. |
| SF-36 [17, 18] | Internal consistency: Cronbach’s alpha values were 0.78-0.93 for subscales [18]. | Content validity: Medical Outcome Study previous literature and wider literature review on instruments [17]. |

References

1. Hodgkinson, K., et al., *The development and evaluation of a measure to assess cancer survivors' unmet supportive care needs: the CaSUN (Cancer Survivors' Unmet Needs measure).* Psycho‐Oncology: Journal of the Psychological, Social and Behavioral Dimensions of Cancer, 2007. **16**(9): p. 796-804.

2. Boyes, A., A. Girgis, and C. Lecathelinais, *Brief assessment of adult cancer patients' perceived needs: development and validation of the 34‐item Supportive Care Needs Survey (SCNS‐SF34).* Journal of evaluation in clinical practice, 2009. **15**(4): p. 602-606.

3. Campbell, H.S., et al., *Psychometric properties of cancer survivors’ unmet needs survey.* Supportive Care in Cancer, 2011. **19**(2): p. 221-230.

4. Hall, A., et al., *Unmet needs of Australian and Canadian haematological cancer survivors: a cross‐sectional international comparative study.* Psycho‐Oncology, 2013. **22**(9): p. 2032-2038.

5. Hall, A., et al., *Factors associated with haematological cancer survivors experiencing a high unmet need across multiple supportive care items: a cross-sectional survey study.* Supportive Care in Cancer, 2014. **22**(11): p. 2899-2909.

6. Campbell, H.S., et al., *Development and validation of the Short-Form Survivor Unmet Needs Survey (SF-SUNS).* Supportive Care in Cancer, 2014. **22**(4): p. 1071-1079.

7. Taylor, K., M. Bulsara, and L. Monterosso, *Test-Retest Reliability of the Short-Form Survivor Unmet Needs Survey.* Asia-Pacific Journal of Oncology Nursing, 2018. **5**(2): p. 165-171.

8. Herdman, M., et al., *Development and preliminary testing of the new five-level version of EQ-5D (EQ-5D-5L).* Quality of Life Research, 2011. **20**(10): p. 1727-1736.

9. Aaronson, N.K., et al., *The European Organization for Research and Treatment of Cancer QLQ-C30: a quality-of-life instrument for use in international clinical trials in oncology.* J Natl Cancer Inst, 1993. **85**(5): p. 365-76.

10. Hjermstad, M.J., et al., *Test/retest study of the European Organization for Research and Treatment of Cancer Core Quality-of-Life Questionnaire.* Journal of Clinical Oncology, 1995. **13**(5): p. 1249-1254.

11. Cella, D.F., et al., *The Functional Assessment of Cancer Therapy scale: development and validation of the general measure.* J Clin Oncol, 1993. **11**(3): p. 570-579.

12. Hlubocky, F.J., et al., *The Development and Validation of a Measure of Health-Related Quality of Life for Non-Hodgkin’s Lymphoma: The Functional Assessment of Cancer Therapy—Lymphoma (FACT-Lym).* Lymphoma, 2013. **2013**: p. 147176.

13. Zebrack, B.J., et al., *Assessing the impact of cancer: development of a new instrument for long‐term survivors.* Psycho‐Oncology: Journal of the Psychological, Social and Behavioral Dimensions of Cancer, 2006. **15**(5): p. 407-421.

14. Crespi, C.M., et al., *Refinement and psychometric evaluation of the impact of cancer scale.* JNCI: Journal of the National Cancer Institute, 2008. **100**(21): p. 1530-1541.

15. Crespi, C.M., et al., *Measuring the impact of cancer: a comparison of non-Hodgkin lymphoma and breast cancer survivors.* Journal of Cancer Survivorship, 2010. **4**(1): p. 45-58.

16. Ferrell, B.R., K. Hassey Dow, and M. Grant, *Measurement of the quality of life in cancer survivors.* Quality of Life Research, 1995. **4**(6): p. 523-531.

17. Ware, J.E., Jr. and C.D. Sherbourne, *The MOS 36-item short-form health survey (SF-36). I. Conceptual framework and item selection.* Med Care, 1992. **30**(6): p. 473-83.

18. McHorney, C.A., et al., *The MOS 36-item Short-Form Health Survey (SF-36): III. Tests of data quality, scaling assumptions, and reliability across diverse patient groups.* Medical care, 1994: p. 40-66.
