# Supplementary material for: Content comparison of unmet needs self-report measures for lymphoma cancer survivors: A systematic review
Source: PLoS One. 2023 Dec 15;18(12):e0290729. doi: 10.1371/journal.pone.0290729 (PMC10723710; doi:10.1371/journal.pone.0290729)
Supplement: Attachment — Submitted filename: Response to Reviewers.docx [file pone.0290729.s004.docx]

Journal Requirements

| 1. Please ensure that your manuscript meets PLOS ONE's style requirements, including those for file naming. The PLOS ONE style templates can be found at  <https://journals.plos.org/plosone/s/file?id=wjVg/PLOSOne_formatting_sample_main_body.pdf> and  <https://journals.plos.org/plosone/s/file?id=ba62/PLOSOne_formatting_sample_title_authors_affiliations.pdf> | Noted and implemented. |
| --- | --- |
| 2. Please include your tables as part of your main manuscript and remove the individual files. Please note that supplementary tables (should remain/ be uploaded) as separate "supporting information" files | Tables included as the main manuscript and removed as individual files. “Supporting information” files amended accordingly. |
| 3. Please include captions for your Supporting Information files at the end of your manuscript, and update any in-text citations to match accordingly. Please see our Supporting Information guidelines for more information: <http://journals.plos.org/plosone/s/supporting-information>. | Noted and implemented. |

| Reviewer 1 | |
| --- | --- |
| Introduction   1. You should add that several instruments have been studied to increase quality of life of patients with cancer in different settings (doi: 10.3390/nu14142958 - doi: 10.1007/s00520-020-05869-0), expecially during COVID-19 pandemic (https://doi.org/10.1186/s44158-022-00067-2). Please briefly discuss and add these 3 references. | We thank you for your time, effort and expertise in reviewing our manuscript. Please find a detailed response to your review below.   1. Thank you for the suggestion, the connection between the suggested papers and the research manuscript needs further clarification as a clear link is not currently understood. |
| Methods   1. All systematic reviews should be registered in PROSPERO before starting the systematic search. Did you perform a registration? Please insert the registration number or add it as a limitation of the study. 2. Please modify Table 1 into PICOS format (intervention and control must be underlined). | 1. This is noted, many thanks. Added to limitations. 2. Table 1 modified into PICOS format. |
| Results   1. Please explain in the results section why a meta-analysis was not feasible. | 1. Updated, pp. 7.   “Statistical pooling of results for meta-analysis was not performed as there were not enough studies or sufficiently similar studies to allow results to be combined (1).” |
| Discussion   1. Please add a conclusion section. | 1. Conclusion pp. 12.   “Standardisation in selecting outcome measurement instruments in specific research areas is warranted as it contributes to improved consistency in reporting and reduces difficulties in comparing and synthesising findings (22). There is a continued need to work in partnership with lymphoma survivors to ensure that future care is responsive to the concerns of this population. Like other tumour-site-specific research, lymphoma survivors, a substantially increasing cohort, will benefit from focused research. Healthcare professionals endeavour to ensure that physical or mechanical tools (i.e., a sphygmomanometer) provide accurate information with each use (i.e., correct blood pressure reading). This concern should be granted to the use of measurement instruments about their validity and reliability in research and broader clinical practice. However, selecting outcome measures for specific purposes is complex, involving conceptual considerations (i.e., defining the construct and population), practical aspects (i.e., the burden for patients and costs) and quality aspects assessed by different measurement properties. Further studies are warranted to assess the measurement properties of existing instruments, especially for structural validity, cross-cultural validity and reliability.  This review provides a platform for instrument comparisons for adult lymphoma survivors, with suggestions for important factors to consider in the systematic selection of unmet needs and quality of life self-report measures. Primarily focused on the research approach, this review has provided steps to consider for clinical applications. In selecting measurement instruments, researchers should consider research objectives, study design, psychometric properties, the instrument’s feasibility and the pros and cons of using more than one measure.” |

| Reviewer 2 | |
| --- | --- |
| Introduction   1. Emphasize the importance of measuring cancer survivors' unmet needs and quality of life outcomes. 2. Clarify the focus on lymphoma cancer survivors and the need for tumor site-specific research. 3. Explain the challenges in selecting appropriate instruments for measuring outcomes. 4. State the objectives of the systematic review. 5. Precision treatment of post-pneumonectomy unilateral laryngeal paralysis is crucial for patients who have undergone cancer or thyroid surgery. This approach involves targeted therapies and interventions, such as laryngeal reinnervation or vocal fold injections, to restore vocal fold function and improve patient outcomes. Tailoring the treatment to each individual's specific needs enhances the effectiveness and ensures optimal recovery of vocal function and quality of life.please discuss and cite doi:10.2217/fon-2019-0053 | We thank you for your time, effort and expertise in reviewing our manuscript. Please find a detailed response to your review below.   1. As the number of cancer survivors continues to rise, it is important that healthcare services and policymakers understand this population’s needs and quality of life outcomes (5). This information can focus on the most problematic or prevalent concerns for cancer survivors or facilitate the detection of problems that might otherwise be overlooked (6). 2. Paragraphs 2 and 3 focus on the lymphoma survivors and the need for site-specific research. 3. Selecting appropriate instruments for measuring outcomes of interest is challenging given the vast proliferation of such questionnaires over the past decades (7), which have been associated with a general lack of clarity in their intended applications (6, 8). 4. Objectives defined on pp. 4.   The objectives are:   i) To identify available instruments measuring the unmet needs and quality of life outcomes of lymphoma survivors in current literature. ii) To conduct a content comparison of the included instruments and appraise the pertinent psychometric measurement properties. iii) To provide evidence-based recommendations for applying patient-reported lymphoma-specific outcome measurements in future research. 5. Thank you for the suggestion, but the connection between this particular concern and the research manuscript needs further clarification as it appears to be outside the scope of this research. |
| Methods   1. Organize the methods section with subheadings for study design, search strategy, eligibility criteria, conceptual framework and content analysis, and assessment of measurement properties. 2. Simplify descriptions of the search strategy and eligibility criteria. 3. Describe the Supportive Care Framework for Cancer Care and its application in the content analysis. | 1. Subheadings as described within the methods section, PP. 4 -6. 2. Search strategy & eligibility criteria revised, PP. 5 - 6. 3. SCF described and its application in the content analysis, i.e., “Individual mapping of the content of items of the included instruments against the framework’s domains was conducted to illustrate the fundamental domains of unmet needs addressed by each instrument.” PP. 6. |
| Results   1. Present a summary of the identified instruments and their psychometric properties. 2. Discuss the content comparison of instruments based on the Supportive Care Framework for Cancer Care. | 1. Summary of identified instruments and their psychometric properties – Table 2. 2. Content comparison based on SCF revised, PP. 11. |
| Discussion   1. Provide evidence-based recommendations for selecting appropriate instruments for future research. 2. Vocal outcomes after cervical surgery, particularly thyroid and parathyroid procedures, can vary depending on the surgical techniques employed and the preservation of the recurrent laryngeal nerve. Minimally invasive and nerve-sparing approaches are often utilized to reduce the risk of vocal fold paralysis and voice dysfunction. Meticulous surgical planning, intraoperative neuromonitoring, and surgeon expertise play crucial roles in ensuring optimal vocal outcomes and minimizing complications postoperatively, please discuss and cite DOI:10.23812/19-282-L | 1. Evidence-based recommendations for future research in the context of selecting appropriate instruments, PP. 13 – 15. 6. Same as Introduction, Point 5. Thank you for the suggestion, but the connection between this particular concern and the research manuscript needs further clarification. It appears to be outside the scope of this research. |
| Conclusion   1. Summarize the findings and their implications for lymphoma cancer survivor research. 2. Emphasize the importance of selecting appropriate instruments for measuring unmet needs and quality of life outcomes. 3. Suggest areas for future research and improvement in the field. | 1 – 3. Thank you for your valuable insight, these considerations have been implemented within the conclusion. |
